# The New Medical Education Law

**Published:** 1889-07

**Authors:** 


### *THE NEW MEDICAL EDUCATION LAW.*

The following bill has been signed by the Governor, and has, therefore, become a law:

### AN ACT

TO PROVIDE FOR THE PRELIMINARY EDUCATION OF MEDICAL STUDENTS.

*The People of the State of New York, represented in Senate and Assembly, do enact as follows:*

SECTION 1. Before the regents of the University of the State of New York, or the trustees of any medical school or college within this

State, shall confer the degree of doctor of medicine on any person who has not received a baccalaureate degree in course from a college or university duly authorized to confer the same, they shall require him to file with the Secretary or recording officer of their university or college, a certificate showing that, prior to entering upon the prescribed three years' study of medicine, he passed an examination conducted under the authority and in accordance with the rules of the Regents of the University of the State of New York, in arithmetic, grammar, geography, orthography, American history, English composition and the elements of natural philosophy, and such certificate shall be signed by the Secretary of the Regents and countersigned by the principal or commissioner conducting said examination.

SEC. 2. This act shall not apply to persons who have already entered upon the prescribed three years' study of medicine, nor shall it alter the time of study or the courses of medical instruction required to be pursued in the medical colleges of this State by existing statutes.

SEC. 3. This act shall take effect immediately.

This act seeks to aid the cause of higher medical education by inquiry into the preliminary qualifications of students oi medicine, and is perhaps as great a step in that direction as could be expected under the present politico-medical jugglery of the "sects" and the legislators. A trial will soon develop its strength or weakness. It fails to provide against incompetent men, who may receive diplomas from colleges outside this State where these safeguards are not required, settling and practising medicine in New York. This appears to us as a defect, but we shall see.

